# Neuroimaging of Vermiform Giant Arachnoid Granulations in Children

**DOI:** 10.3390/children11070763

**Published:** 2024-06-24

**Authors:** Oswaldo A. Guevara Tirado, Livja Mertiri, Stephen F. Kralik, Nilesh K. Desai, Thierry A. G. M. Huisman, Maarten H. Lequin, Huy (Brandon) D. Tran

**Affiliations:** 1 Ponce Health Sciences University School of Medicine, 388 Zona Industrial Reparada 2, Ponce, PR 00716, USA; oguevara20@stu.psm.edu; 2 Edward B. Singleton Department of Radiology, Texas Children’s Hospital and Baylor College of Medicine, 6701 Fannin Street, Suite 470, Houston, TX 77030, USA; lxmertir@texaschildrens.org (L.M.); sfkralik@texaschildrens.org (S.F.K.); nkdesai@texaschildrens.org (N.K.D.); huisman@texaschildrens.org (T.A.G.M.H.); mxlequin@texaschildrens.org (M.H.L.)

**Keywords:** arachnoid granulations, vermiform giant arachnoid granulations, pediatric radiology, neuroimaging, dural sinus thrombosis

## Abstract

Arachnoid granulations (AGs) are generally benign structures within the subarachnoid space that extend into the dural sinuses and calvarial bone. They can present in a variety of sizes but are termed ‘giant’ arachnoid granulations (GAGs) when they are larger than 1 cm in diameter or take up a significant portion of the dural sinus’ lumen. Vermiform giant arachnoid granulations are a specific type of GAG that are known for their worm-like appearance. Specifically, these vermiform GAGs can be challenging to diagnose as they can mimic other pathologies like dural sinus thrombosis, sinus cavernomas, or brain tumors. In this case series, we present two cases of vermiform giant arachnoid granulations, discuss their imaging characteristics and highlight the diagnostic challenges to improve identification and prevent misdiagnoses.

## 1. Introduction

Arachnoid granulations (AGs) or Pacchionian granulations, named after the Italian physician Antonio Pacchioni (1665–1726) are typically benign, collagen and immune cell-rich structures within the subarachnoid space that extend into the dural sinuses and calvarial bone [1,2,3,4,5]. These formations exhibit a wide range of sizes and morphologies and are typically best visualized in MRI studies after 10 years of age [3,6]. Despite a lack of consensus in the literature, they are typically termed ‘giant’ arachnoid granulations (GAGs) when their size exceeds 1 cm in diameter, or they occupy a significant portion of a dural sinus’ lumen [1,2,3,4,5,7]. Within the spectrum of GAGs exist the vermiform giant arachnoid granulations, characterized by their distinctive worm-like appearance [2,3]. Because of their variability in presentation, AGs and GAGs present a unique diagnostic challenge as they can mimic pathologies such as dural sinus thrombosis, sinus cavernoma, and tumors like meningiomas or metastatic invasions of the sinus [1,2,3,8]. This case report aims to present and elucidate the imaging characteristics and differential diagnoses of vermiform GAGs to assist clinicians in accurately identifying these formations and prevent both misdiagnoses and improper management.

## 2. Cases

**Figure 1:**
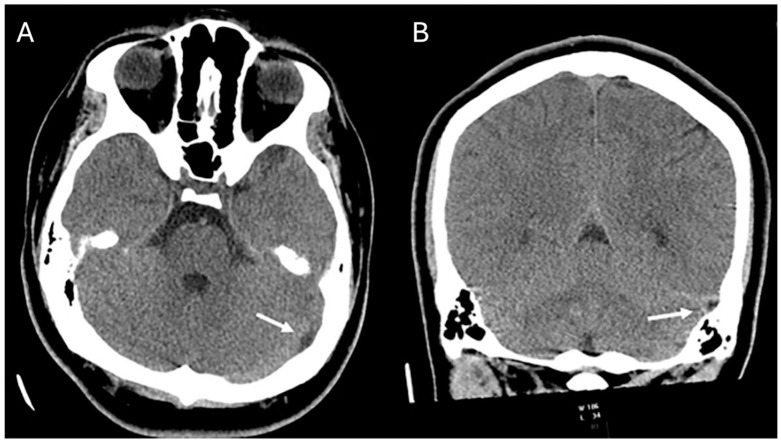
Axial (**A**) and coronal (**B**) CT images demonstrate a hypodense area along the course of the left sigmoid and transverse sinus (arrows) compatible with a CSF filled Pacchionian granulation extending into the lumen of the affected dural sinuses.

**Figure 2:**
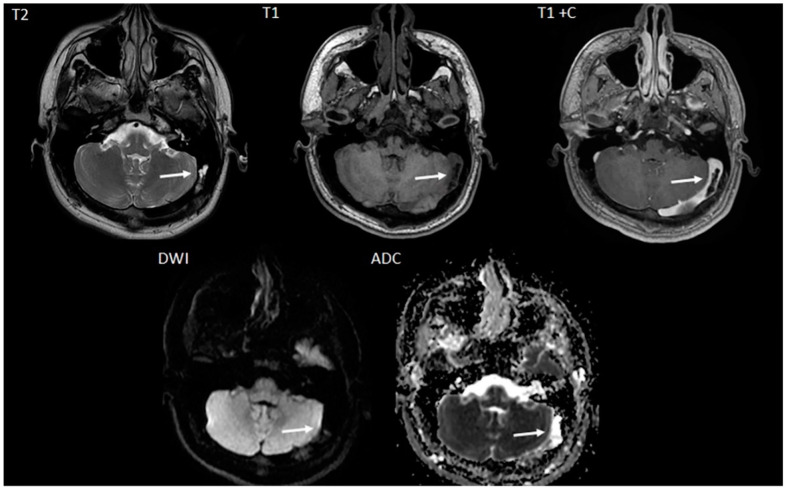
Axial T2-weighted (T2), T1-weighted (T1), T1-weighted with contrast (T1+C), diffusion weighted image (DWI) MRI images, and apparent diffusion coefficient (ADC) map reveal a CSF isointense, T2-hyperintense giant arachnoid granulation matching the CT finding (arrows). On the contrast enhanced sequence, contrast enhancement is noted outlining the non-enhancing Pachionic granulation. On diffusion weighted imaging (DWI) and the matching apparent diffusion coefficient (ADC) map the Pacchionian granulation is characterized by facilitated diffusion (DWI-hypointense, ADC bright) similar to CSF.

**Figure 3:**
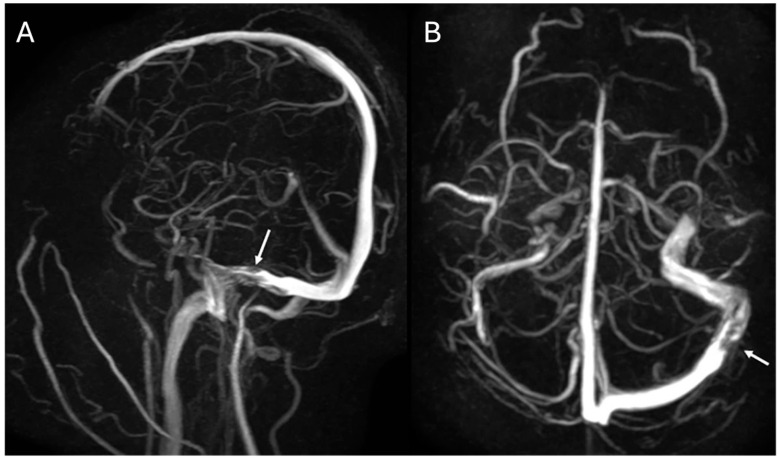
Sagittal (**A**) and axial (**B**) Maximum Intensity Projection (MIP) MR-venography images display a filling defect (arrows) within the patent left distal transverse and proximal sigmoid sinus region consistent with an arachnoid granulation extending into the patent dural sinuses.

The first patient was a 15-year-old male who presented with a history of chronic, recurrent headaches. An initial computed tomography (CT) scan (Figure 1) was performed and upon interpretation a concern for a dural sinus thrombosis was voiced. Thus, a follow-up brain magnetic resonance imaging (MRI) (Figure 2) was performed which revealed a T2-hyperintense signal within the course of the left transverse/sigmoid sinus. This finding was compatible with an arachnoid granulation. This lesion further demonstrated high apparent diffusion coefficient (ADC) values which confirmed the cystic and fluid nature of the lesion. T1-weighted contrast-enhanced images also showed a matching endoluminal filling defect. MR venography (Figure 3) revealed normal flow in the left dural sinus which circumvented the arachnoid granulation. The MRI characteristics were diagnostic of a vermiform giant arachnoid granulation. A dural sinus thrombosis could be excluded for certainty.

**Figure 4:**
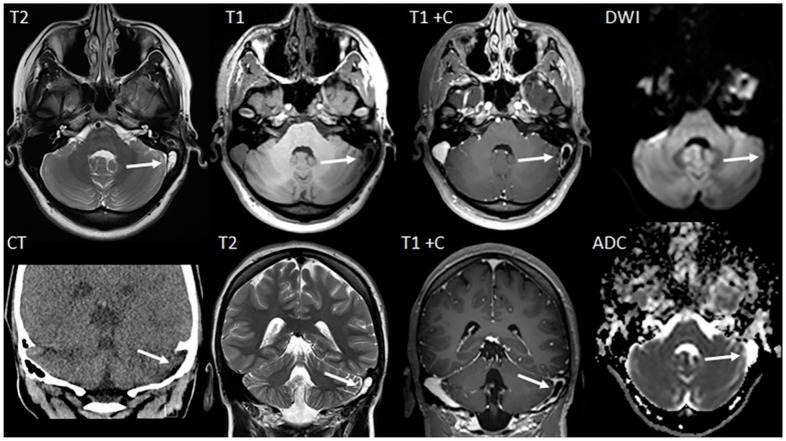
Axial MR images (T2-weighted, T1-weighted, T1-weighted with contrast, and DWI images) and matching coronal CT and MRI images (T2-weighted, T1-weighted with contrast, and axial ADC map) show a CSF-isointense, T2-hyperintense, T1-hypointense lesion in the left transverse/sigmoid sinus region (arrows). On the contrast enhanced T1-weighted sequence the lesion does not enhance. On DWI the lesion is DWI-hypointense with facilitated diffusion characteristics on the matching ADC map. The CT scan identifies this lesion as hypodense, while the MRI sequences confirm the diagnosis of a giant arachnoid granulation while excluding an endoluminal/dural sinus thrombus.

The second patient was a 14-year-old female patient who presented with a history of chronic headaches and vision problems. The initial CT scan demonstrated a hypodense area within the region of the left sigmoid sinus (Figure 4). Further evaluation with MRI (Figure 4) demonstrated a T2-hyperintense tubular appearing arachnoid granulation that extended into the left sigmoid sinus. Diffusion-weighted imaging (DWI) and matching ADC maps showed facilitated diffusion (high ADC values) confirming the cystic and fluid nature of the lesion. This finding was key to excluding a solid thrombus as on the contrast-enhanced T1-weighted MRI, the lesion resulted in an apparent contrast filling defect. Without the T2-weighted MR images and DWI/ADC characteristics, the lesion might have been incorrectly diagnosed as a dural sinus thrombus.

## 3. Discussion

Arachnoid granulations (AGs) are membrane outgrowths that play a crucial role in cerebrospinal fluid (CSF) reabsorption [1,3,6,9]. They present in various shapes and sizes and are often incidentally noted in imaging studies of the transverse and superior sagittal sinus [1,2,3,4,5,6,7,8,9]. Giant arachnoid granulations (GAGs), especially their vermiform subtype, pose a unique diagnostic challenge due to their distinctive morphology, often resembling serious pathologies like dural sinus thrombosis, sinus cavernoma, and tumors [1,2,3]. Understanding the differing characteristics of AGs, GAGs, and the mimicking pathologies is thus essential for accurate diagnosis as well as optimal patient management and outcomes.

Specifically, failure to accurately distinguish AGs and GAGs from a differential like dural sinus thrombosis can have devastating consequences for a patient. Such misdiagnosis could lead to unwarranted invasive procedures or the initiation of anticoagulation therapy [1,7]. Starting anticoagulation therapy unnecessarily may inadvertently cause intracranial bleeding, which can result in additional medical interventions, prolonged hospital stays, financial damages, and an overall increase in both morbidity and mortality [1,7].

### 3.1. Symptomatology

Regarding symptomatology, AGs and GAGs are typically asymptomatic and considered benign entities. When they do present with symptoms, patients primarily report with a history of headache, sensory change, and symptoms consistent with intracranial hypertension [2,3,4,5,6,7,8]. At this point, it appears very challenging for a clinician to distinguish an AG or GAG from more common pathologies such as ischemia, thrombosis, or brain tumors based on symptomatology alone. Due to this ambiguity, we turn towards the imaging characteristics of each of these.

### 3.2. Imaging

Before discussing the differing characteristics between AGs, GAGs, and mimicking pathology, it is necessary to review and be familiar with the available imaging studies and sequences. This is especially important for MRI studies as it is the standard of care for visualizing and evaluating brain pathologies [10]. Some of the most commonly used sequences in brain MRI imaging include: T1-weighted, T2-weighted, fluid-attenuated inversion recovery (FLAIR), DWI, and susceptibility-weighted imaging (SWI). Each sequence fulfills a unique role that ranges from evaluating overall structure to assessing the movement or diffusion of water [10]. Understanding and choosing the appropriate sequences is as crucial to getting the correct diagnosis as is formulating an accurate interpretation of the study.

### 3.3. AGs & GAGs

In imaging studies such as CT and MR, arachnoid granulations (AGs) and their giant variants (GAGs) appear as low-density lesions on CT, and as CSF-isointense focal lesions bulging/extending into the dural sinuses on MR [1,2,6,7,8,9,11,12,13]. They commonly appear as filling defects within a dural sinus and lack contrast enhancement [1,2,5,6,7,13,14]. Specifically, they give low signal on T1-weighted MR imaging, and high signal on T2-weighted imaging [8,11,13]. The size of AGs is variable, with GAGs usually exceeding 1 cm in diameter or occupying a significant portion of the sinus lumen [2,8]. These formations exhibit a wide range of morphologies, including nodular, round-to-ovoid, irregular, and vermiform shape [9]. These distinct presentations aid in their differentiation from other pathologies such as tumors or thrombi, which typically show different signal intensity and enhancement patterns [8,11,12,13]. Furthermore, a dural sinus thrombus or a tumor extending into the dural sinus typically presents with reduced diffusion characteristics (DWI-hyperintense with matching elevated ADC values).

### 3.4. Tumors

Brain tumors are typically best evaluated via MRI as it allows us to determine characteristics such as location, extent of involvement, mass effect, vascularity, cellularity, surrounding edema, and areas of hypoxia [10]. These imaging features are useful for differentiating between tumor subtypes as well as from differing pathology. For example, a combination of cystic and solid components might suggest a tumor-like pilocytic astrocytoma, while calcifications may be seen in ependymomas [10]. Signal intensity and enhancement can vary between tumor subtypes. For example, malignancies such as meningiomas or metastasis typically demonstrate higher signal intensity and enhancement due to their vascular nature, while some gliomas may exhibit little to no enhancement [10]. DWI/ADC can also be helpful as ADC values tend to be lower in higher-grade tumors [15]. Ultimately, it is the ability to incorporate all of these characteristics together that allows for optimal differentiation between a malignancy and an AG or GAG.

### 3.5. Thrombi

Thrombi on the other hand may exhibit varied signal characteristics based on their age and stage [15]. This variation in conjunction with their high mortality rates poses a greater diagnostic challenge. Typical MRI findings of a dural sinus thrombosis include iso- or hyperintensity on T1-weighted imaging and hyperintensity on T2-weighted imaging [3]. Another way to differentiate a thrombus from an AG or GAG is via contrast-enhanced MR venography [3]. In addition, a thrombus can be easily differentiated from a cerebrospinal fluid (CSF) filled arachnoid granulation on diffusion weighted imaging as arachnoid granulations are characterized by facilitated diffusion (low DWI-signal intensity and high ADC values) while a thrombus typically shows restricted diffusion [16]. Finally, morphology can be an additional contributing factor towards making the correct diagnosis. Thrombi involve a large portion of a sinus, while AGs can present as smaller, nodular, well-defined lesions [1,2,3]. However, one must not over-rely on this strategy as GAGs have the potential to be just as large and may be easily confused. Thus, it is the presence or absence of blood flow in addition to MR intensities that ultimately lead us to the correct diagnosis.

### 3.6. Other

In addition to considering tumors and thrombotic events, it is critical to account for artifacts, anatomical asymmetries, and structural variations such as septations when interpreting MR venography results [17]. Artifacts, notably signal saturation effects and flow gaps, can misleadingly suggest the absence of flow. Saturation effects, often resulting from slow or in-plane blood flow, may erroneously appear as flow voids [17]. Similarly, flow gaps might be observed as lack of flow on MRV, despite their presence on source images and traditional spin-echo MRI scans [17]. Anatomic asymmetry, particularly from underdeveloped (hypoplastic) or absent (aplastic) transverse sinuses, can lead to misinterpretations of vessel size and flow, with MR imaging potentially underestimating the luminal diameter, especially in smaller or slowly flowing venous sinuses [17]. Furthermore, septations within the sinuses can create linear filling defects, more readily identified on contrast-enhanced MR venography [17].

### 3.7. Limitations & Importance

Despite the advancements in neuroimaging, there are still some limitations when it comes to accurately diagnosing AGs and GAGs. The nonspecific symptomatology, the existence of the previously discussed artifacts, and the variability in granulation morphology all play a significant role in causing misinterpretations. Additionally, the true prevalence of GAGs in living individuals has not yet been investigated via imaging studies [2]. For these reasons it is crucial for both radiologists and physicians to be aware of the imaging characteristics of AGs and GAGs, as well as recognize the importance of using additional imaging sequences on MRI to assist in diagnostic confirmation.

## 4. Conclusions

Filling defects within the dural sinuses can result from a multitude of factors, ranging from benign entities like arachnoid granulations and artifacts to life-threatening conditions such as dural sinus thrombosis and tumors. Accurate identification of vermiform giant arachnoid granulations (GAGs) from the more serious pathologies is crucial to ensure correct patient management and avoid unnecessary interventions. This report highlights the importance of maintaining vermiform GAGs as a differential diagnosis and presents two cases within the typical age range where arachnoid granulations can be seen serving as a reference for similar cases. Further research, including prospective follow-up studies should be performed to understand long-term outcomes, underlying mechanisms for granulation development, and optimal management strategies for patients with vermiform GAGs.

## Data Availability

All data is contained within the article.
